# Potential Mechanisms for Traditional Chinese Medicine in Treating Airway Mucus Hypersecretion Associated With Coronavirus Disease 2019

**DOI:** 10.3389/fmolb.2020.577285

**Published:** 2020-12-14

**Authors:** Yuanfeng Zhang, Zheyi Wang, Yue Zhang, Hongxuan Tong, Yiling Zhang, Tao Lu

**Affiliations:** ^1^School of Life Sciences, Beijing University of Chinese Medicine, Beijing, China; ^2^Department of Encephalopathy, Dongzhimen Hospital, Affiliated to BUCM, Beijing, China; ^3^Institute of Basic Theory for Chinese Medicine, China Academy of Chinese Medical Sciences, Beijing, China

**Keywords:** coronavirus disease 2019, airway mucus, mucins, expectorant formulae, network pharmacology, traditional Chinese medicine

## Abstract

**Background:**

The rapid development of coronavirus disease 2019 (COVID-19) pandemic has become a great threat to global health. Its mortality is associated with inflammation-related airway mucus hypersecretion and dysfunction of expectoration, and the subsequent mucus blockage of the bronchioles at critical stage is attributed to hypoxemia, complications, and even death. Traditional Chinese medicine (TCM) has rich experience in expectorant, including treatment of COVID-19 patients with airway mucus dysfunction, yet little is known about the mechanisms. This study is aiming to explore the potential biological basis of TCM herbal expectorant for treating COVID-19.

**Objective:**

To get core herbs with high used frequency applications in the actions of expectoration by using association rule algorithm and to investigate the multitarget mechanisms of core herbs in expectorant formulae for COVID-19 therapies.

**Methods:**

Forty prescriptions for expectorant were retrieved from TCM Formulae. The ingredient compounds and targets of core herbs were collected from the TCMSP database, Gene-Cards, and NCBI. The protein interaction network (PPI) was constructed by SRING, and the network analysis was done by Cytoscape software. Bioconductor was applied for functional enrichment analysis of targets.

**Results:**

The core herbs of expectorant could regulate core pathways (MAP kinase activity, cytokine receptor binding, G-protein-coupled receptor binding, etc.) via interactions of ingredients (glycyrol, citromitin, etc.) on mucin family to eliminate phlegm.

**Conclusion:**

TCM herbal expectorant could regulate MAPK and cytokine-related pathways, thereby modulating Mucin-family to affect mucus generation and clearance and eventually retarding the deterioration of COVID-19 disease.

## Introduction

The recent pandemic of pneumonia caused by a novel coronavirus has been rampant across the world, with the number of victims increased daily (Coronavirus Disease, 2020; Li L. Q. et al., 2020; Mahase, 2020; Petrosillo et al., 2020); so far, over 30 million have been infected and nearly 1 million succumbed globally, a mortality rate approaching 3.1% according to WHO. Coronavirus disease 2019 (COVID-19) has resulted in more deaths than severe acute respiratory syndrome (SARS) and Middle East respiratory syndrome (MERS) combined (Mahase, 2020). An early analysis (Huang et al., 2020) on 41 cases confirmed at the initial stage of the epidemic (diagnosed until January 2) showed that, except for the common symptoms such as fever, cough, myalgia, or fatigue, sputum production (28%) was the most common atypical symptom. It was seen from another analysis (Guan et al., 2020) on 1,099 confirmed cases (diagnosed until January 29) that sputum production (33.7%) was the fourth most common symptom following fever (88.7%), cough (67.8%), and fatigue (38.1%). Recently, an autopsy analysis (National Health Commission of the People’s Republic of China and National Administration of Traditional Chinese Medicine, 2020) on the patients who succumbed to coronavirus due to hypoxemia and respiratory distress has revealed that a large number of viscous secretions could be found seeping from the air sacs, white foamy mucus adhering in the airway, and gelatinous mucus adhering in the bronchial lumen. Much mucus with high viscosity instigating small airway (lack of goblet cells) obstruction was the most prominent feature of COVID-19, which leads to infections, ventilation dysfunction, acute respiratory distress syndrome (ARDS), and uncorrectable hypoxemia. Overproduction of mucins interacts with or forms aggregates with platelets and might lead to the plugging of pulmonary airways (Bikdeli et al., 2020; Deshpande, 2020; Lax et al., 2020). Therefore, the abnormal secretion of airway mucus was one of the major causes of death in critical patients.

In the lungs of healthy people, mucus is barely secreted and constantly cleared by ciliated epithelium via expectoration or being swallowed into the throat. High mucus secretion is usually a clinical feature of severe respiratory disease. When a viral infection and a subsequent inflammation occurs, loads of filamentous actin (F-actin), neutrophil-derived DNA, dead cells, and cellular debris are accumulated to sequester pathogens, forming mucous purulence, often called phlegm after being coughed up (Voynow and Rubin, 2009). The pathological examination results of COVID-19 were similar to those of SARS (Xu et al., 2020) but showed a main difference in patients with COVID-19 who suffered from distal alveolar injury (Liu et al., 2020) with significant exudative reaction (Hoffmann et al., 2020). This is attributed to much of the mucus with high viscosity adhering and obstructing the small airways. The current clinical treatments for COVID-19 include oxygen reception (38.0%), mechanical ventilation (6.1%), intravenous antibiotics (57.5%), and oseltamivir (35.8%) (Guan et al., 2020). While the mechanical ventilation is an important treatment measure, its curative effect is seriously impacted as the viscous mucus is distributed deep inside the air sacs and bronchioles, thereby impeding expectoration due to ventilation pressure. It has been reported that guaifenesin (a mucus diluent) and about 6 L of normal saline were used to clear the mucus in the airway for COVID-19 patients (Holshue et al., 2020). These treatments were used for directly adding water or simulating water secretion in the respiratory tract to increase the hydration of the phlegm (Rubin, 2007) and, consequently, to dilute the phlegm. Bromhexime (a mucus-dissolving agent) inhibiting the synthesis of respiratory glands and goblet cells has also been used (Malerba and Ragnoli, 2008). It can reduce viscosity by generating acid mucopolysaccharides to secrete small molecular mucin with less mucus (Medical Encyclopedia, 2016). Abnormal mucus secretion compromises the ventilation functions, thereby preventing the ventilator oxygen from reaching the air sacs in severe patients. From the above, how to clear the mucus in the small airway becomes an urgent issue to be solved.

The traditional Chinese medicine (TCM) has rich experiences in combating epidemic over the past thousands of years. In fact, the mortality rate of COVID-19 has been notably reduced in many regions of China after the implementation of TCM, thanks to its remarkable achievements in preventing the deterioration into severe or critical stage (State Administration of Traditional Chinese Medicine, 2020). From TCM prescriptions in the Diagnosis and Treatment Plan for COVID-19 issued by the National Health Commission, as well as the “Qing-Fei-Pai-Du Decoction” recommended by the State Administration of TCM, we found that phlegm-eliminating herbs or compatibles were used frequently but in different dosage. In this study, we assessed prescriptions with phlegm-eliminating effects via evaluating their ingredient components and potential targets. We aim to (Li L. Q. et al., 2020) obtain the high-frequency herb combinations with the expectorant effects, (Mahase, 2020) reveal the synergistic molecular mechanisms of the expectorants used in the treatment of COVID-19 through the network pharmacology, and (Petrosillo et al., 2020) provide scientific evidences for preventing the deterioration of COVID-19 pneumonia into severe and critical stage after the combination of TCM and western medicine.

## Data and Methods

### Analysis on Medication Rules of Expectorant Formulae

**FIGURE 1:**
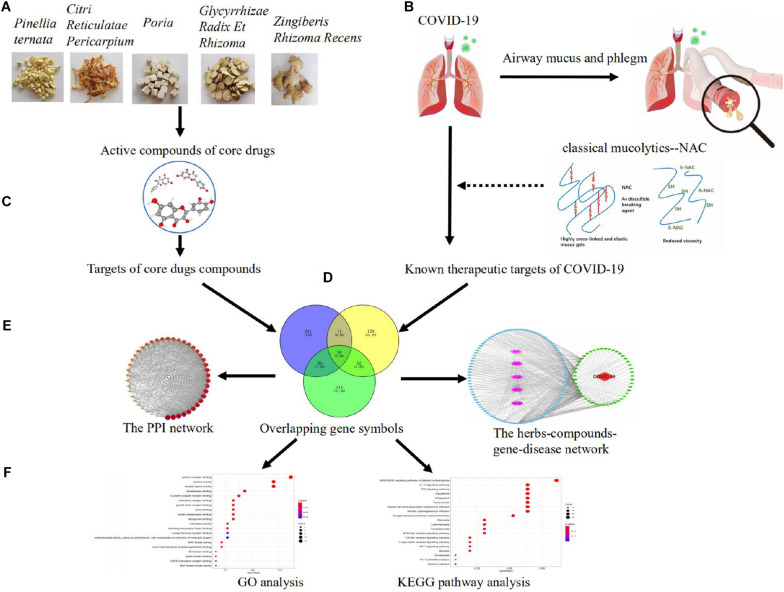
Pharmacological mechanisms of eliminating phlegm from mucus hypersecretion caused by COVID-19. Illustration of experimental workflow: **(A)** five high-frequently used herbs obtained by using association rule algorithm; **(B)** data collection, the collection of compounds and targets of high-frequently used herbs; **(C)** construction of known compound–target network; **(D)** detection of COVID-19 and phlegm disease target modules and overlapping analysis; **(E)** PPI network of high-frequently used herbs and COVID-19 intersecting targets, as well as the herbs–compounds–targets–diseases network; **(F)** analysis of GO function and KEGG pathways.

The whole workflow is illustrated in Figure 1.

### Establishment of Database

A total of 40 expectorant prescriptions (329 herbs) were obtained from the *Formula of TCM* (Deng, 2008); a final 113 herbs with relatively high record frequency (average >2) were exported for this study.

#### Analysis on Herb-Usage Frequency for Expectorant

Herb-usage frequency analysis was carried out for the expectorant formulae, and herbs were ranked according to their usage frequency.

#### Analysis on High-Frequently Used Herbs

On the basis of the analysis on herb-usage frequency of expectorant formulae, the support degree was set to 15% (the frequency of the data is at least 15% of all data) to obtain the high-frequently used herbs of the expectorant formulae by using the association rule algorithm, and displayed in Cytoscape3.7.2.

### Molecular Mechanism of Core Herbs

#### Screening of Major Active Compounds of Core Herbs and Determination of Action Targets Thereof

In this study, the major active compounds of five Chinese herbs (Citri Reticulatae Pericarpium, Poria, Glycyrrhizae Radix Et Rhizoma, Zingiberis Rhizoma Recens, *Pinellia ternata*), which are the most commonly used herbs of the expectorant formulae, were searched out from the Traditional Chinese Medicine Systems Pharmacology Database (TCMSP)^1^. Oral bioavailability (OB) greater than or equivalent to 30% and drug-likeness (DL) greater than or equivalent to 0.18 were set to be the screening parameters (Guo et al., 2019; Lee W. Y. et al., 2019; Xu et al., 2012). The action targets of such major compounds were also searched from this database, and the obtained target protein information was standardized by using Uniport database^2^. Consequently, the gene numbers of the key targets corresponding to the compounds in such five herbs were obtained.

#### Target Collection and Potential Target Prediction of COVID-19

The targets of COVID-19 were searched from Gene-Cards database^3^ and NCBI database, and the related genes of COVID-19 were retrieved by using “*Homo sapiens*” as species and “novel coronavirus” as keywords. The predicted targets of the herbs and the targets of the disease were mapped by using Venny2.1 mapping software so as to obtain the potential action targets of the core herbs of the expectorant formulae in the treatment of COVID-19.

#### Construction of PPI Network and Herbs–Compounds–Targets–Disease Network

The potential targets were constructed to be a target protein interaction (PPI) network through the search tool for the retrieval of interacting genes/proteins (STRING^4^). The Cytoscape3.7.2^5^ was used for constructing the network of herbs–compound components–action targets–disease.

#### Enrichment Analysis on Target Functional Pathway

The analysis on the function enrichment and the main action pathway of the core target genes was carried out by using Bioconductor^6^ to perform Gene Ontology (GO) enrichment and Kyoto Encyclopedia of Genes and Genomes (KEGG) pathway analysis and visualize the results of the enrichment analysis.

## Results

### Frequency of Commonly Used Herbs From Expectorant Formulae

According to statistics of expectorant formulae, the most frequently used is Banxia (*P. ternata*); of the 329 involved Chinese herbs, the top 20 ranked with relatively high usage frequency are as shown in Table 1.

**TABLE 1:** Usage frequency of herbs in expectorant formulae.

No.	Chinese name	Latin name	Frequency
1	Ban Xia	*Pinellia ternata*	30
2	Chen Pi	Citri Reticulatae Pericarpium	24
3	Fu Ling	Poria	22
4	Gan Cao	Glycyrrhizae Radix Et Rhizoma	21
5	Sheng Jiang	Zingiberis Rhizoma Recens	15
6	Tian Nan Xing	Arisaematis Rhizoma	10
7	Zhi Shi	Aurantii Fructus Immaturus	9
8	Huang Qin	Scutellariae Radix	7
9	Huang Lian	Coptidis Rhizoma	7
10	Bei Mu	Fritillariae Cirrhosae Bulbus	7
11	Ku Xing Ren	Armeniacae Semen Amarum	6
12	Xiang Fu	Cyperi Rhizoma	5
13	Jie Geng	Platycodonis Radix	5
14	Zhu Ru	Bambusae Caulis In Taenias	4
15	Zhi Qiao	Aurantii Fructus	4
16	Zhi Zi	Gardeniae Fructus	4
17	Shi Chang Pu	Acori Tatarinowii Rhizoma	4
18	Jiang Can	Bombyx Batryticatus	4
19	Gua Lou ZI	Trichosanthis Semen	4
20	Dang Gui	Angelicae Sinensis Radix	4

### Analysis on Prescription-Formulating Principles Based on Association Rule Algorithm

**FIGURE 2:**
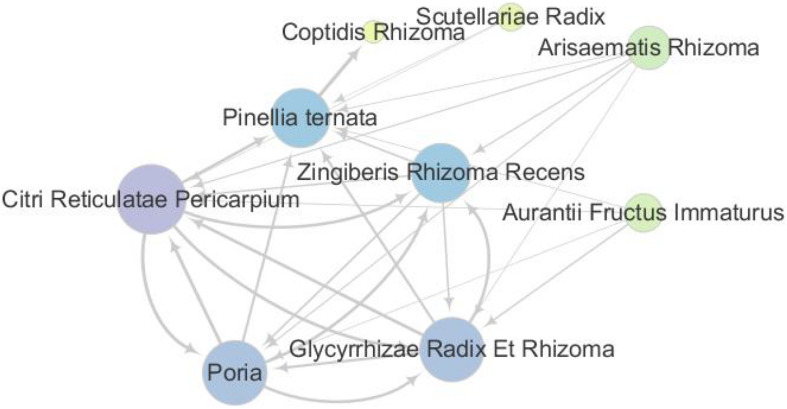
Network of common combinations in formulae. The herbs concurrent usage for patients with phlegm. The 40 prescriptions (329 herbs) were analyzed through the association rules. Citri Reticulatae Pericarpium, Poria, Glycyrrhizae Radix Et Rhizoma, Zingiberis Rhizoma Recens, *Pinellia ternata*, Aurantii Fructus Immaturus, Arisaematis Rhizoma, and Scutellariae Radix Coptidis Rhizoma were the most commonly used herbs among Chinese herbal formulae.

The associations between the herbs were obtained by analyzing the association rules for the data. The herbs combinations with support degree >15% were selected so as to obtain the reliability of the data. Then, those selected data were sorted from high to low frequency. The sorted result showed that the associations and the frequencies between Citri Reticulatae Pericarpium, Poria, Glycyrrhizae Radix Et Rhizoma, Zingiberis Rhizoma Recens, and *P. ternata* were higher than those between other herbs and, consequently, showed that such herbs were the core herbs of the expectorant formulae (as shown in Table 2 and Figure 2).

**TABLE 2:** Frequency of core combination in *formulae*.

No.	Rules	Frequency	Support	No.	Rules	Frequency	Support
1	*Pinellia ternata* ⇒ Citri Reticulatae Pericarpium	21	0.52	12	Citri Reticulatae Pericarpium ⇒ Arisaematis Rhizoma	9	0.23
2	Pinellia ternata ⇒ Poria	19	0.47	13	Pinellia ternata ⇒ Aurantii Fructus Immaturus	9	0.23
3	*Pinellia ternata* ⇒Glycyrrhizae Radix Et Rhizoma	19	0.47	14	*Pinellia ternata* ⇒Arisaematis Rhizoma	9	0.23
4	*Pinellia ternata* ⇒ Poria	18	0.45	15	Poria ⇒ Arisaematis Rhizoma	8	0.2
5	Citri Reticulatae Pericarpium ⇒ Glycyrrhizae Radix Et Rhizoma	18	0.45	16	*Pinellia ternate* ⇒ Scutellariae Radix	7	0.18
6	Glycyrrhizae Radix Et Rhizoma ⇒ Poria	15	0.38	17	Citri Reticulatae Pericarpium ⇒ Aurantii Fructus Immaturus	7	0.18
7	*Pinellia ternata* ⇒Zingiberis Rhizoma Recens	15	0.38	18	Citri Reticulatae Pericarpium ⇒ Scutellariae Radix	6	0.15
8	Zingiberis Rhizoma Recens ⇒ Glycyrrhizae Radix Et Rhizoma	11	0.28	19	Poria ⇒Aurantii Fructus Immaturus	6	0.15
9	Poria ⇒ Zingiberis Rhizoma Recens	11	0.28	20	Glycyrrhizae Radix Et Rhizoma ⇒ Arisaematis Rhizoma	6	0.15
10	Citri Reticulatae Pericarpium ⇒ Zingiberis Rhizoma Recens	11	0.28	21	HuangLian ⇒ *Pinellia ternata*	6	0.15
11	Glycyrrhizae Radix Et Rhizoma ⇒ Aurantii Fructus Immaturus	9	0.23	22	Zingiberis Rhizoma Recens ⇒ Arisaematis Rhizoma	6	0.15

For the herb display with 15% of support degree, the frequency of occurrence was represented by the scatter size and the color gradation [color gradation value range, min 1.0 (yellow) to max 10.0 (purple)], and the degrees of association between the herbs were represented by the thickness of the lines.

According to the frequency of the herb combination, which were ranked from high to low, the top 5 herbs are *P. ternata*, Citri Reticulatae Pericarpium, Poria, Glycyrrhizae Radix Et Rhizoma, and Zingiberis Rhizoma Recens. The pharmacological actions of high-frequency usage herbs are listed in the *Pharmacopoeia of the* People’s Republic of China (National Pharmacopoeia Committee, 2005) and *Chinese Medicine* (Zhong, 2016), shown in Table 3.

**TABLE 3:** Pharmacological action of the five core herbs.

Core herb name	Pharmacological actions
*Pinellia ternata*	Eliminating phlegm, inhibiting the secretion of gastric juice, increasing the activity of tyrosine transaminase, promoting bile secretion, antivomiting, and antitumor effect (Du et al., 2019)
Citri Reticulatae Pericarpium	Relaxing bronchial smooth muscle, relieving asthma, alleviating cough, relaxing intestinal smooth muscle tension, antioxidant, anti-inflammatory, antigastric ulcer, promoting gastric emptying, and regulating intestinal movement (Ni et al., 2019)
Poria	Increasing myocardial contractility, enhancing immunity, antifibrotic (Chen et al., 2020), diuretic, sedative, and antitumor effects (Shi et al., 2017); hypoglycemic, antiaging, antigastric ulcer, and liver protection (Ríos, 2011; Kim et al., 2019)
Glycyrrhizae Radix Et Rhizoma	Regulating immunity, antibacterial, antiviral, anti-inflammatory (Lee S. A. et al., 2019), antiallergic, antiulcer, spasmolytic, liver protection, antitussive and expectorant, antidotal, antiarrhythmia, antitumor (Li J. et al., 2020), antitissue fibrosis, antihyperlipidemic, antiatherosclerosis, and inhibiting platelet aggregation (Jiang et al., 2020)
Zingiberis Rhizoma Recens	Stimulating vascular motor/respiratory center, promoting the secretion of digestive juice, protecting gastric mucosa, antiulcer, anti-inflammation, antipyretic, antibacterial, analgesic, antiemetic, antimycobacterial and immune modulating (Bhaskar et al., 2020)

During the coronavirus pandemic, the National Health Commission of the People’s Republic of China issued the “Diagnosis and Treatment Protocol for Novel Coronavirus Pneumonia (Trial Version),” which is based on the accumulating experience in the diagnosis and treatment. Qingfei Paidu Decoction (QFPD) was one of the most recommended Chinese medicine prescriptions, encompassing all five core herbs (Ban Xia, Chen Pi, Fu ling, Sheng jiang, Gan Cao). The clinical trials with QFPD and others are shown in Table 4.

**TABLE 4:** The therapeutic effects of the five core herbs involving remedies.

Area	Sample size	Study type	Clinical efficacy
Sichuan (Wang E.-C. et al., 2020)	75	Observational study	QFPD has shown good clinical efficacy in the treatment of COVID-19, which can significantly improve the laboratory indicators (AST, ALT, LDH, CK, α-hydroxy butyric acid dehydrogenase, *p* < 0.05)
Hubei (Li H. et al., 2020)	749	Retrospective Study	The application of TCM can reduce the hospital stay, duration for medication, and the time needed for negative diagnosis of the disease, facilitating the disappearance of some main clinical symptoms for COVID-19 and effectively improving the outcome of chest CT.
Shang hai (Xin et al., 2020)	63	Retrospective Study	The combination treatment of COVID-19 with QFPD and Western Medicine could relieve the symptoms and alleviate inflammation in the lung and mitigate the extent of multiorgan impairment in patients with COVID-19
Sichuan (Wang R. Q. et al., 2020)	98	Observational study	Above 90% curative rate based on clinical lab results (LYM, AST, ALT, D-dimer), with no deterioration to severe stage
Henan (Jiansheng et al., 2016)	364	Interventional study	In patients with dyspnea, sputum purulence, and sputum amount syndrome diagnosed as acute exacerbations of COPD, TCM treatments have significant benefit in relieving symptoms (dyspnea, etc.) and improving quality of life

Moreover, a large-scale report of 1,265 patients from 10 provinces has 98% curative rate with no conversion to severe stage, according to the National Administration of Traditional Chinese Medicine (National Administration of Traditional Chinese Medicine, 2020). There are few ones against COVID-19 with TCM remedy that encompasses all five herbs, which are listed in Chinese Clinical Trial Registry^7^, shown in Table 5.

**TABLE 5:** The ongoing clinical trials of the five core herbs involving remedy.

Area	Sample size	Study type	Registration number
Beijing	782	Observational study	ChiCTR2000032767
Sichuan	100	Observational study	ChiCTR2000030883
Wuhan	20	Observational study	ChiCTR2000030806

### Screening of the Active Compounds of the Core Herbs and the Action Target Results

**FIGURE 3:**
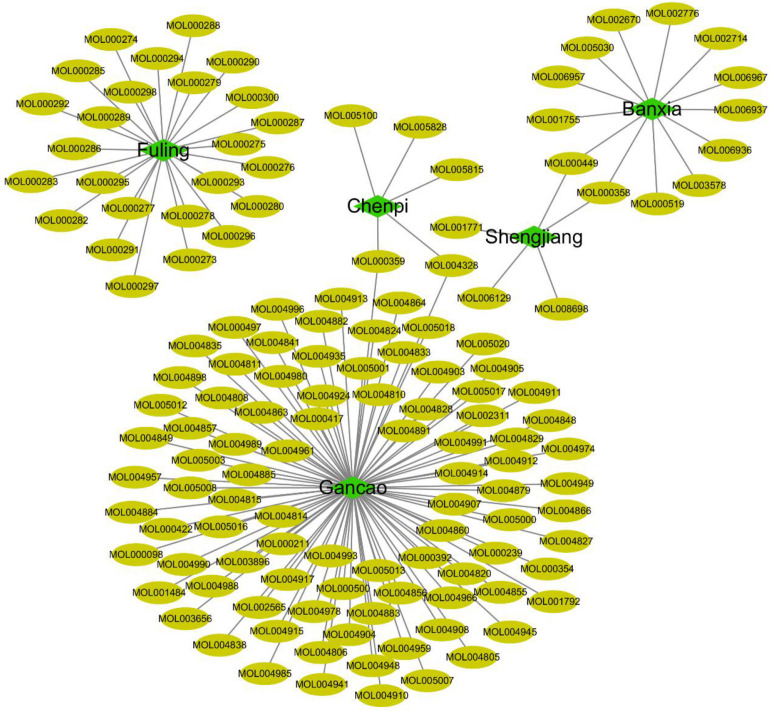
Compounds from core herbs functioning in eliminating phlegm. Green nodes represent five high-frequently used herbs. Yellow nodes represent active candidate compounds shown as entry names, such as MOL000297 from Fuling (Poria) is Cerevisterol. All the detailed information of the active compound was shown in Supplementary Table.

In this study, 758 compounds were obtained from Citri Reticulatae Pericarpium, Poria, Glycyrrhizae Radix Et Rhizoma, Zingiberis Rhizoma Recens, and *P. ternata* by TCMSP platform. One hundred forty main active compounds were screened according to the conditions of OB greater than or equivalent to 30% and DL greater than or equivalent to 0.18. Within all the 140 main active compounds, 13 were from *P. ternata*, 5 from Citri Reticulatae Pericarpium, 25 from Poria, 92 from Glycyrrhizae Radix Et Rhizoma, and 5 from Zingiberis Rhizoma Recens (as shown in Figure 3). The active compounds were displayed in the Supplementary Table according to the OB value. Then, the regulatory target information of the main active compounds of TCM was searched from TCMSP database to obtain 227 corresponding action targets. Finally, the targets and the gene information were standardized by Uniprot.

### Prediction of Potential Targets of the Core Herbs of the Expectorant Formulae in Treatment of COVID-19 and Phlegm Production

**FIGURE 4:**
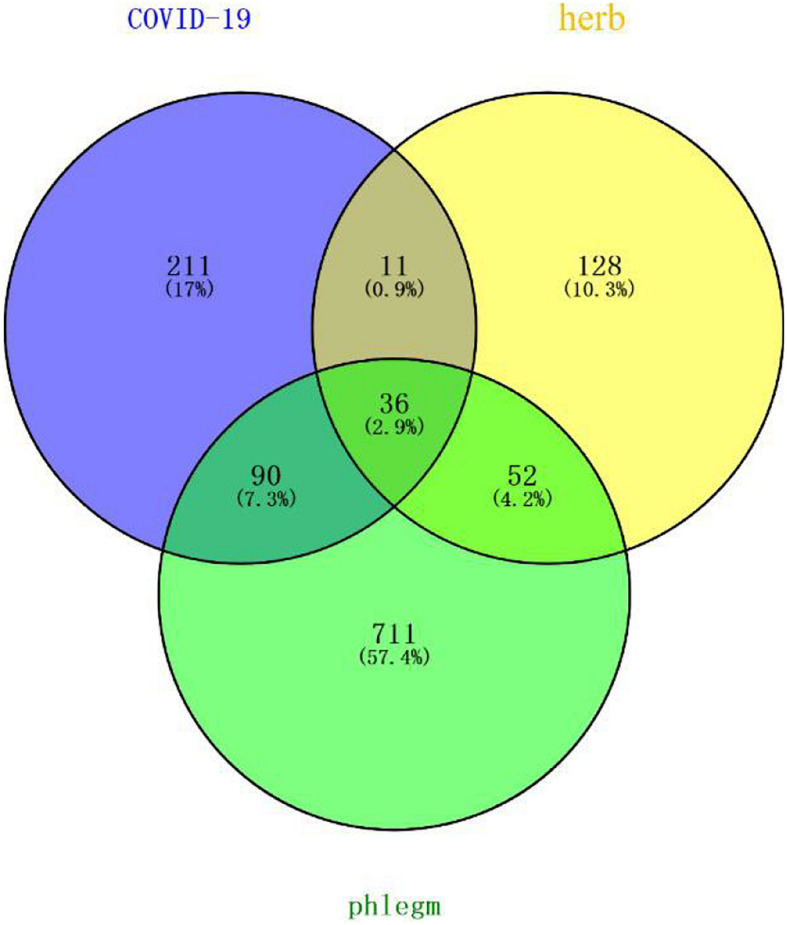
Potential targets of core herbs in the treatment of phlegm secretion caused by COVID-19. Purple circle represents targets of COVID-19. Yellow circle represents targets of herbs. Green represents targets of phlegm. There were 36 overlapping genes among the COVID-19 and phlegm and herbs.

Forty-eight and 346 potential targets of COVID-19 were respectively searched from NCBI and Gene-Cards to obtain 348 related targets of COVID-19 after removing 46 repetitions. The targets regulated by the main active compounds of the core herbs of the expectorant formulae were intersected with the treatment targets of COVID-19 by Venny2.1 to obtain 47 targets. Eight hundred eighty-nine targets were obtained by searching “phlegm” and “sputum” (191 results of phlegm, 815 results of sputum, and the repeated ones were removed) in Gene-Cards. Thirty-six of them were intersected with the obtained 47 targets in the treatment of COVID-19 (as shown in Figure 4).

### Construction of the Interaction Network of Core Herbs of the Expectorant Formulae and the Protein of COVID-19

**FIGURE 5:**
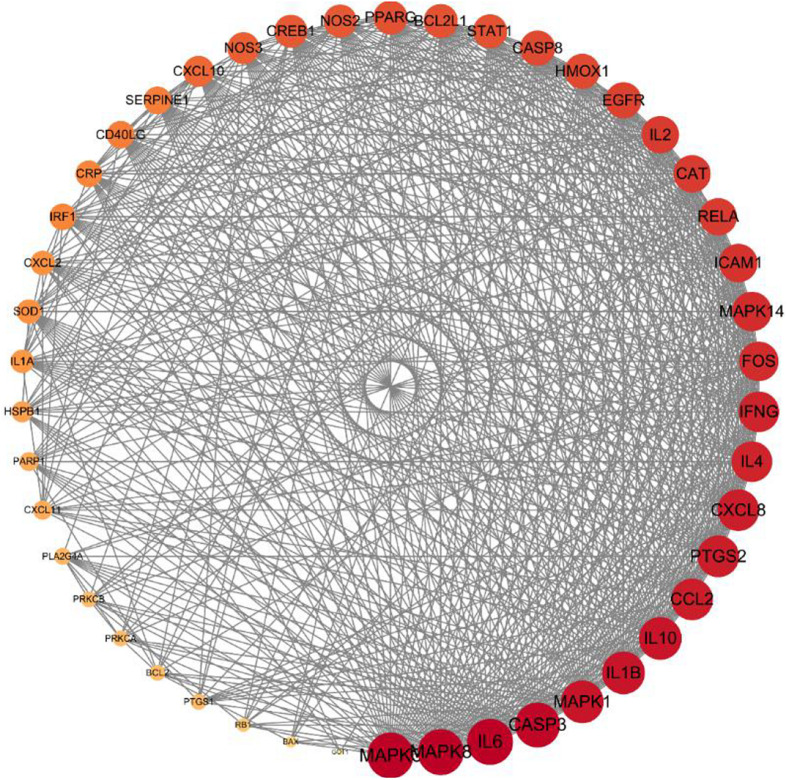
Core gene targets for eliminating phlegm related to COVID-19. Protein–protein interaction (PPI) network of intersecting targets of core components and phlegm. More lines, related to greater degree of association; larger nodes, correlated to higher degree of the nodal value.

The PPI network analysis, carried out on the STRING platform, obtained 47 intersection genes from the mapping of the herbs and the disease. A network diagram (as shown in Figure 5) was obtained after importing the data into Cytoscape3.7.2. Forty-seven nodes (representing proteins) and 605 edges (representing the interactions between the proteins) were included in the diagram. The average node degree value of the target proteins was 25.7, and 28 target proteins had higher node degree. The degree values of MAPK3, MAPK8, IL-6, CASP3, MAPK1, IL-1β, IL-10, CCL2, PTGS2, CXCL8, IL-4, IFNG, FOS, MAPK14, ICAM1, RELA, CAT, IL-2, EGFR, HMOX1, CASP8, STAT1, BCL2L1, PPARG, NOS2, CREB1, NOS3, and CXCL10 were significantly greater than that of other targets, and such 28 targets played an important role in connections in the PPI network. The core herbs of the expectorant formulae could be used for treating COVID-19 through the above targets.

### The Herbs–Compounds–Targets–Disease Network

**FIGURE 6:**
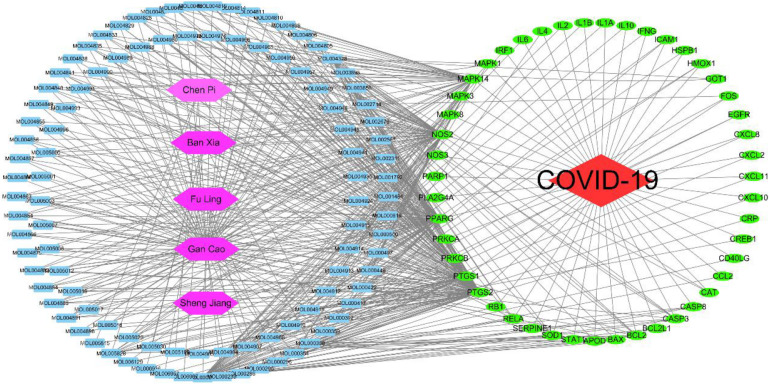
Network of herbs–compounds–targets–disease. Interactions between components are connected with lines. Red node represents disease; purple nodes represent the herbs; the blue nodes stand for the main active compounds of the core herbs from the phlegm eliminating recipes; the green nodes typify the potential targets; and each line stand for the interaction between a compound and a target. In this network, the degree of each node represents the number of lines connected with the nodes; core nodes were screened according to characteristics of network topology, such as the node degree value. Active compounds are shown as entry names, such as MOL005018 from Gan Cao (Glycyrrhizae Radix Et Rhizoma) is Xambioona. All the detailed information for active compound is shown in the Supplementary Table.

The herbs–compounds–targets–disease network (as shown in Figure 6) was constructed by using Cytoscape3.7.2. One hundred fifty-five nodes were included. The screening result showed the relationships and connections among the compounds and the action targets. The compounds, such as quercetin, naringin, beta-sitosterol, kaempferol, nobiletin, Glycyrrhizae Radix Et Rhizoma chalcone A, scutellarin, formononetin, and isorhamnetin, might be the core compounds of the core herbs in the expectorants. The action targets, such as PTGS2, PPARG, NOS2, PTGS1, MAPK14, BCL2, CASP3, RELA, BAX, MAPK1, SOD1, CASP8, and PRKCA, played the key role in the treatment of COVID-19.

Thirty-six common targets, including MAPK14, SERPINE1, IL2, NOS2, CASP3, CD40LG, ICAM1, PARP1, IL1A, PTGS2, PRKCA, MAPK1, CXCL11, CAT, EGFR, BCL2, NOS3, BCL2L1, RELA, PPARG, CREB1, PTGS1, SOD1, MAPK8, HSPB1, and CXCL2, of the phlegm-eliminating herbs in the treatment of COVID-19 and phlegm productions were found out in Gene-Cards, wherein the MAPK family and the inflammatory factors were close to the top of the list.

### Mucin Production

Normal mucus is made up of 97% water and 3% solids, which are mainly made up of mucin, nonmucin proteins, salts, lipids, and cell debris. MUC5AC and MUC5B were strongly expressed in the airways. Among them, mucin is regulated at the molecular level in a variety of ways; although the pathways that connect them are not yet fully established, EGFR receptor signal transduction and inflammatory factors are involved in regulating the mucin.

**FIGURE 7:**
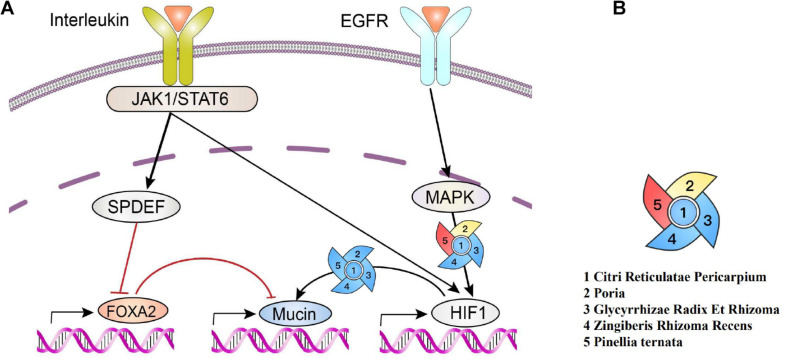
Production of polymeric mucins and their regulations by herbs. **(A)** Interleukin activates JAK1/Stat6, leading to the increase in SPDEF expression and subsequently inhibiting of FOXA2 expression, which downregulates mucin. Meanwhile, EGFR activates MAPK, and HIF-1 can also be activated via signal cascades from EGFR and JAKI/STAT6, which upregulates mucin. **(B)** Red indicates a positive effect (Wang et al., 2019). Blue indicates a negative effect (Zhao Dong-mei and Tao, 2015; Park et al., 2016; Li et al., 2017; Lu et al., 2018; Choi et al., 2019; Jiang et al., 2019; Zhai et al., 2019; Zhang et al., 2020). Yellow indicates a bidirectional regulation (Wang et al., 2018; Choi et al., 2019).

Inflammatory factors, including IL-13, could activate JAKI, cause the phosphorylation of STAT6, and produce positive and negative feedback regulations on mucin. EGFR-activated MAPK and hypoxia-inducible factors (HIF-1) regulate the production of mucin MUC5A (as shown in Figure 7).

### Analysis on Target Pathways

**FIGURE 8:**
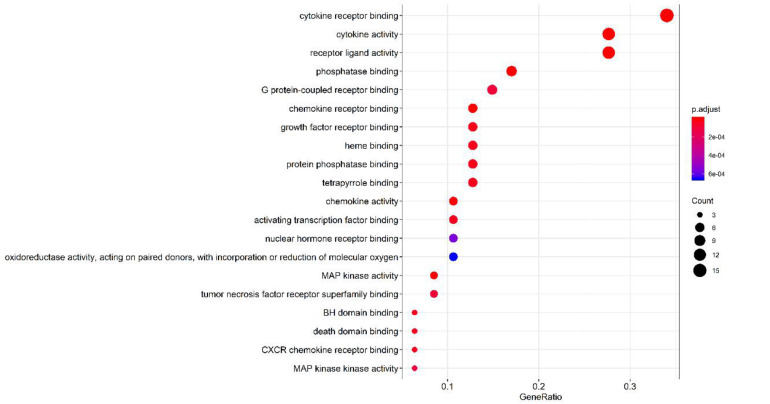
Top 20 pathways of the core targets from GO enrichment analysis (bubble chart). *X*-axis represents significant enrichment in the counts of these terms. *Y*-axis represents the categories of “biological process” in the GO of the target genes (*p* < 0.01).

The GO function enrichment analysis was carried out through a bioconductor to obtain 71 items of biological processes (BP) (*p* < 0.01), wherein a histogram and a bubble chart were drawn for the top 20 GO items (as shown in Figure 8). The target enrichment was relatively concentrated in the biological processes, such as cytokine receptor binding, cytokine activity, receptor ligand activity, chemokine receptor binding, phosphatase binding, MAP kinase activity, chemokine activity, growth factor receptor binding, heme binding, protein phosphatase binding, BH domain binding, death domain binding, tetrapyrrole binding, CXCR chemokine receptor binding, activation transcription factor binding, tumor necrosis factor receptor superfamily binding, G-protein-coupled receptor binding, MAP kinase activity and nuclear hormone receptor binding. The result revealed that the active compounds of the core herbs of the phlegm eliminating recipe had effects on airway mucus hypersecretion caused by COVID-19 through regulating various biological pathways.

**FIGURE 9:**
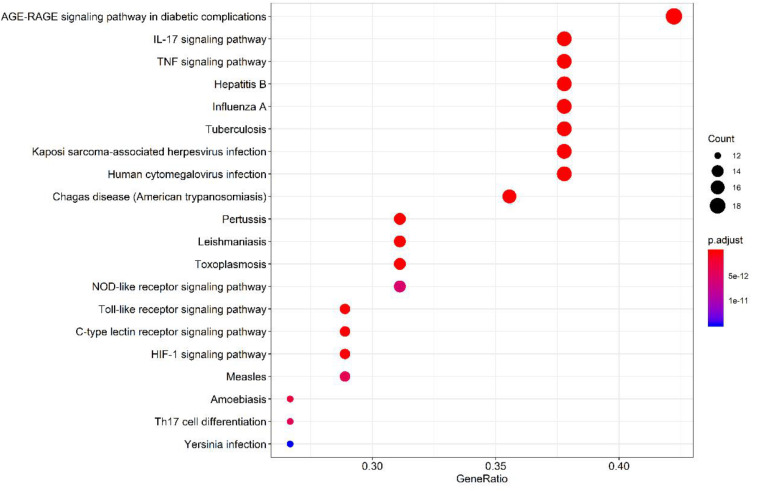
Top 20 pathways of the core targets from KEGG enrichment analysis (bubble chart). *X*-axis represents the counts of the target symbols in each pathway; *Y*-axis represents the main pathways (*p* < 0.01).

Of the 131 signal pathways that were screened from KEGG pathway enrichment (*p* < 0.01), 20 with relatively lower *p* values were selected (as shown in Figure 9). These genes were enriched more in the pathways, such as diabetic complication AGE-RAGE signaling pathway, IL-17, and TNF signaling pathway and C-type lectin receptor signaling pathway. Eight of the 20 pathways were related to virus infections, including pertussis, leishmaniasis, hepatitis B, influenza A, tuberculosis, Kaposi’s sarcoma-related herpesvirus infection, human cytomegalovirus infection, and measles. It revealed that the core herbs of the phlegm-eliminating recipe played an important role in antiviral and anti-inflammatory fields, which might be the reason for using them in the treatment of COVID-19.

## Discussion

### Potential Mechanisms of TCM in Treating Airway Mucus Hypersecretion

COVID-19 invades cells via the angiotensin-converting enzyme II (ACE2) receptor, the same host cell receptor for SARS-CoV, except for much high binding affinity of the S-protein (Hoffmann et al., 2020). In this study, network analysis was carried out for the compounds in the core combinations of the phlegm-eliminating herbs and the targets against COVID-19. The Cytoscape3.7.2 was used for constructing the herbs–compounds–targets–diseases network, and the result showed that the action targets played a key role in the whole network, such as PTGS2 (COX2), PTGS1 (COX1), MAPK14, PPARG, NOS2, BCL2, CASP3, RELA, BAX, MAPK1, SOD1, CASP8, and PRKCA. The transcription level of mucin was highly regulated, involving a critical role of HIF-1 in the regulation of Muc5ac. The activation of EGFR-mediated signaling pathways leads to HIF-1α production and HIF-1 activation, resulting in mucin expression in human airway epithelial cells (Young et al., 2007; Yu et al., 2012). Moreover, EGFR signal inhibitors could inhibit the increase in Muc5ac through MAPK or HIF-1 (Burgel and Nadel, 2008; Kettle et al., 2010); and the core herbs of the phlegm-eliminating herbs were discussed having the effects similar to that of the EGFR receptor signal inhibitors. All herbs have downregulating effects on HIF-1; Citri Reticulatae Pericarpium, Glycyrrhizae Radix Et Rhizoma, and Zingiberis Rhizoma Recens have downregulating effects on MAPK, except for *P. ternata*. Poria has downregulating effects on MAPK (Figure 7 EGFR–MAPK–mucin) but has upregulating effects on MAPK when applied to the skin.

It could be seen from the results of network pharmacology analysis that the compounds, such as quercetin, naringenin, beta-sitosterol, kaempferol, nobiletin, licochalcone A, scutellarin, formononetin, and isorhamnetin, have the highest comprehensive score of the core herb compounds. They played a key role on the targets that induced the production of mucin, such as MAPK14 and MAPK1. The previous studies revealed that 6-gingerol in ginger (Zingiberis Rhizoma Recens) decreased MUC5A through ERK and MAPK pathways (Kim et al., 2009). Further analysis suggested that MAP kinase activity and nuclear hormone receptor binding were involved.

One hundred thirty-one signal pathways were screened from KEGG pathway enrichment analysis (*p* < 0.01). Target genes were enriched more in IL-17 and TNF signaling pathways, which directly or indirectly regulate the expression of MUC5AC. Moreover, these pathways were related to virus infection, including pertussis, leishmaniasis, hepatitis B, influenza A, tuberculosis, Kaposi’s sarcoma-related herpesvirus infection, human cytomegalovirus infection and measles. Overexpression of inflammatory factors (IL-4, 9, 13, 17, 23, 25) (Hung et al., 2008; Curran and Cohn, 2010; Peng et al., 2010) could increase the expression of MUC5AC and downstream transcription factors. It was speculated that the core herbs of the expectorant formulae played an important role in antiviral, anti-inflammatory, and inhibition of mucin synthesis, suggesting a substantial role in combating COVID-19, especially in warding off mucus plugs-related exacerbation toward severe or critical stage.

### Airway Mucus and Phlegm

The so-called “mucus plugs” or “phlegm plugs” in the anatomical results of COVID-19 patients were formed by the pulmonary mucus lesions. Mucus is a viscous, gel-like material consisting of various macromolecules, inorganic salts, and water. Normal mucus and mucociliary clearance are the key components of lung innate immune function (Rubin, 2014; Poole et al., 2019). Airway mucus maintains hydration in the airway and traps particulates, bacteria, and viruses (Lillehoj et al., 2013). During infection or inflammation, more mucus was secreted in the airway as a result of the enlargement of goblet cells and submucous glands, forming the mucus hypersecretion reaction. The mucus is usually removed by the interaction of air flows and cilia, and the phlegm is mainly removed through coughing. Pathogens and particles are trapped in the inhaled air by the mucus and are removed from the lungs and airways by air flows and cilia; therefore, lung functions will be abnormal if the mucus clearance function is impaired (Fahy and Dickey, 2010). Secretion clearance depends on the mucus properties, such as viscoelasticity, adhesiveness, serous properties, and ciliary functions.

The inflammatory process caused the loss of the functions of cells and cilia, and the airway phospholipase destroyed the surface-active layer and changed the biological and physical characteristics of the surface mucus of the airway. The characteristic gel-like property of mucus is believed to be attributable mainly to the presence of polydisperse glycoproteins or mucins. There are 17 genes in the encoding mucin of the human body. Five of the secreted mucins have terminal cysteine-rich domains that can form disulfide bonds resulting in polymers that impart the properties of a gel. MUC5AC and MUC5B are gel-forming airway mucins and are strongly expressed in the human respiratory tract and display the characteristics of mucociliary clearance and barrier functions. The MUC5AC is an airway mucin produced by the goblet cells of the surface epithelium (Ma et al., 2018), and MUC5B is produced in the mucus cells in the whole airway and the submucosal glands. The ratio of MUC5AC and MUC5B varies with the health status.

Hypersecretion of mucus in the bronchioles and air sacs of the patients with COVID-19 blocks the small airway, which is related to the uncontrolled MUC5B defense mechanism (Roy et al., 2014) and the abnormal secretion of MUC5A. The viscous mucus blocks the bronchioles, thus making the mechanical ventilation effect unsatisfactory (People’s Daily Online, 2020). In patients with moderate-to-severe chronic obstructive pulmonary disease (COPD), long-term use of N-acetylcysteine 600 mg twice daily can prevent exacerbations (Zheng et al., 2014). The favorable effects of N-acetylcysteine (NAC) on acute exacerbations of COPD are attributable to its mucolytic actions (Cazzola and Matera, 2014), similar to the expectorant effect of abovementioned herbs. NAC breaks the disulfide bonds that link mucin monomers to polymers, reducing the viscosity of the sputum, the severity of coughing, and the number of bacteria in the airways (Dekhuijzen, 2006). Over production of mucus leads to morbidity and mortality by obstructing airflow and shielding bacteria from antibiotics. Mucins interact with or form aggregates with platelets (Bhatia et al., 2019) when the immunity is weakened. Platelet-activating factor functions as a mucus secretagogue (Rieves et al., 1992). Furthermore, pulmonary embolism is another major pathological aspect responsible for the death of COVID-19 (Deshpande, 2020; Lax et al., 2020). In this study, we found that the core phlegm-eliminating herbs have downregulating effects on mucin (Figure 6, EGFR-MAPK-mucin), suggesting that it is important to use the phlegm-eliminating herbs against COVID-19.

### Traditional Chinese Medicine Treatment for COVID-19

Facing the challenge of the novel coronavirus pneumonia, the State Administration of TCM initiated the “clinical screening study of effective prescriptions for prevention and treatment of COVID-19” and recommended the use of QFPD. The recipe comprises Citri Reticulatae Pericarpium, *P. ternata*, Poria, and Glycyrrhizae Radix Et Rhizoma, forming the major part of Er-Chen decoction (Xie, 2020). The Er-Chen decoction, a typical expectorant, had the efficacy of removing dampness and resolving phlegm. The data analysis on prescriptions that related to Er-Chen decoction (323 prescriptions) in “*The Dictionary of Traditional Chinese Medicine Prescriptions*” revealed a similar function of eliminating phlegm, removing dampness, along with regulating Qi and strengthening spleen (Huang et al., 2010). Pathogenesis of COVID-19 is complicated, involving multiorgans, such as the lung, spleen, kidney, and stomach. In TCM principles, dampness or phlegm was connected not only to the damage of lung but also to the weakness of spleen (Xie, 2020), which is associated with the status of immunity. Therefore, the herbs for eliminating phlegm and removing dampness were beneficial to the patients of COVID-19 at multiple dimensions. Most of the herbs that we discussed target the lung and spleen (Zhao et al., 2020), having effects on dissolving phlegm, resolving turbidness, dispelling dampness, and facilitating diuretic effect (Tong et al., 2020). Long-term use of mucolytic drug/herbs could prevent mucus hypersecretion exacerbations (Zheng et al., 2014; CINN, 2020; Peking University Team, 2020). Compounds from TCM prescriptions can inhibit the excessive secretion of the airway mucin (Kim et al., 2009; Shin et al., 2013; Liu et al., 2017) via regulating mucin family proteins in treating respiratory diseases with mucus hypersecretion symptom.

Furthermore, other aspects of these herbs, such as regulating the energy (Qi) and immunity status and boosting functions for anti-inflammation and antivirus, provided an integrative solution to combat the COVID-19.

### Summary

In this study, we have systematically screened the expectorant remedies/herbs in TCM, using association rule algorithm analysis and network pharmacology. Top 5 herbs (*P. ternate*, Citri Reticulatae Pericarpium, Poria, Glycyrrhizae Radix Et Rhizoma, and Zingiberis Rhizoma Recens) were most frequently used and highly related to expectorant. Interestingly, these top 5 herbs were coincidently a part of the 21 herbs of QFPD decoction and being a major part of Er-Chen decoction, the typical expectorant TCM remedy. QFPD decoction has been proven effective in the remission of COVID-19 infection. The QFPD was specifically designed to treat major symptoms from COVID-19, including expectorant. However, other functions of QFPD, e.g., anti-inflammation, antiviral, immunity-boosting, etc., have been mechanistically investigated (Zhong et al., 2020), except for expectorant. In this study, we provided molecular mechanisms for expectorant of these top herbs, focusing on their regulatory mechanisms on mucin related pathways, such as HIF-1/MAPK and interleukin, aiming to confer further insight for TCM attribution to the remission of COVID-19 infection. Further experiments and studies are needed to clarify the synergistic mechanisms of these ingredients.

## Data Availability Statement

All datasets presented in this study are included in the article/Supplementary Material.

## Author Contributions

TL, YfZ, and ZW contributed to conception and design of the study. YfZ and ZW analyzed the data and produced the figures. YfZ wrote the manuscript. YlZ, YZ, and HT contributed to manuscript revision. All authors contributed to read and approved the submitted version.

## Conflict of Interest

The authors declare that the research was conducted in the absence of any commercial or financial relationships that could be construed as a potential conflict of interest.

## Footnotes

1 http://tcmspw.com/tcmsp.php

2 https://uniprot.org/

3 https://www.genecards.org/

4 https://string-db.org/

5 http://www.cytoscape.org/

6 http://www.bioconductor.org/

7 http://www.chictr.org.cn/

## Abbreviations

TCM: traditional Chinese medicine
COVID-19: coronavirus disease 2019
SARS: severe acute respiratory syndrome
MERS: Middle East respiratory syndrome
PER: high-frequency phrase
TCMSP: Traditional Chinese Medicine Systems Pharmacology
NCBI: National Coalition Building Institute
PPI: protein–protein interaction
WHO: World Health Organization
ARDS: acute respiratory distress syndrome
GO: gene ontology
KEGG: Kyoto Encyclopedia of Genes and Genomes
OB: oral bioavailability
DL: drug-likeness
BP: biological processes
MAPK: mitogen-activated protein kinase
EGFR, epidermal growth factor receptor: also for short ErbB-1 or HER1
HIF-1: hypoxia inducible factor-1
NAC: *N*-acetylcysteine.
